# Pituitary volume as a permissive morphological threshold for hypothalamic-pituitary-gonadal axis activation in children: a comprehensive diagnostic prediction study with relative importance decomposition

**DOI:** 10.3389/fendo.2026.1981603

**Published:** 2026-10-22

**Authors:** Jianjian Cai, Ling Zhang, Weiyin Vivian Liu, Qin Liu, Jun Lu, Dong Liu

**Affiliations:** 1 Department of Radiology, Tongji Hospital, Tongji Medical College, Huazhong University of Science and Technology, Wuhan, Hubei, China; 2 Department of Urology, Tongji Hospital, Tongji Medical College, Huazhong University of Science and Technology, Wuhan, Hubei, China; 3 MR Research, GE Healthcare, Beijing, China; 4 Department of Critical Care Medicine, Tongji Hospital, Tongji Medical College, Huazhong University of Science and Technology, Wuhan, Hubei, China

**Keywords:** diagnostic prediction model, hypothalamic-pituitary-gonadal axis, pituitary volume, precocious puberty, Shapley value decomposition

## Abstract

**Objective:**

The gonadotropin-releasing hormone (GnRH) stimulation test, the reference standard for confirming hypothalamic-pituitary-gonadal axis (HPGA) activation, is invasive and costly. Pituitary volume (PV) on MRI has been proposed as a non-invasive surrogate, yet whether PV constitutes a permissive morphological threshold or is redundant with clinical parameters remains undefined. We evaluated PV as a morphological threshold of HPGA activation and quantified its relative importance, unique information content, and clinical utility.

**Methods:**

We retrospectively enrolled 896 children who underwent pituitary MRI and GnRH stimulation testing. PV was measured using the ellipsoid approximation. Out-of-fold cross-validated AUC was the primary performance metric. Predictor importance was quantified by Shapley value decomposition, permutation importance, and Lindeman–Merenda–Gold (LMG) variance partitioning. Morphological-threshold analyses examined HPGA activation across PV deciles. Statistical mediation tested whether insulin-like growth factor 1 (IGF-1) mediated the PV–HPGA association, with formal power analysis for incremental AUC detection.

**Results:**

PV exhibited a threshold pattern: HPGA activation was 28.9% (95% CI 20.5–39.0) in the lowest PV decile (≤115 mm³) versus 92.2% (95% CI 84.8–96.2) in the highest (≥335 mm³; OR 4.38, 95% CI 2.60–7.36; per-IQR OR 2.04, 95% CI 1.59–2.62). PV ranked second by Shapley decomposition (+0.065 AUC, 22.2%), carrying unique information (LMG 20.4% of pseudo-R²). At the 203.9 mm³ cutpoint, the diagnostic odds ratio was 4.78, positive likelihood ratio 2.44, and number needed to screen 2.73. PV tertile AUC was 0.693; combined with IGF-1, AUC rose to 0.748. IGF-1 statistically mediated 37.4% of the PV–HPGA association. The full model OOF AUC was 0.801 (95% CI 0.772–0.828) versus 0.798 for the clinical model (ΔAUC +0.003); power was ~5% at N = 896, requiring N ≈ 25360 for 80% power. The age-sex-adjusted PV z-score AUC was 0.592 (95% CI 0.554–0.630).

**Conclusion:**

PV serves as a permissive morphological threshold for HPGA activation; while it’s not a strictly necessary condition, HPGA activation is physiologically improbable below a critical PV. Although PV is not a standalone predictive tool and its incremental value over clinical parameters is minimal, it functions as a complementary morphological checkpoint that contextualizes hormonal interpretation when pituitary MRI has been conducted to exclude intracranial lesions as per guidelines.

## 1 Introduction

Central precocious puberty (CPP), resulting from premature activation of the hypothalamic-pituitary-gonadal axis (HPGA), affects approximately 1 in 5,000–10,000 children worldwide, with a marked female predominance (1). Early HPGA activation accelerates epiphyseal maturation, compromises adult height, and is associated with psychosocial and metabolic consequences. Timely and accurate diagnosis is therefore essential to guide treatment with gonadotropin-releasing hormone (GnRH) analogs (2). The GnRH stimulation test remains the reference standard for confirming HPGA activation (3, 4). However, it requires serial venipuncture over 60–120 minutes, is costly, and carries a burden of anxiety for children and families. Basal luteinizing hormone (LH) and other biochemical surrogates have been explored, but their diagnostic accuracy varies considerably across assays and populations (5, 6). A reliable, non-invasive biomarker that could triage children before confirmatory testing remains an unmet clinical need.

The pituitary gland undergoes well-characterized morphological changes during normal puberty. Elster et al. (1990) first demonstrated physiological hypertrophy of the adolescent pituitary gland on MRI, showing that gland size increases markedly during puberty, particularly in girls (7). Kato et al. (2002) extended these observations in a large normative series, documenting age- and sex-related morphological remodeling with emphasis on pubescent changes (8). These foundational studies established that pituitary volume (PV) increases progressively with age and pubertal stage, reflecting hypertrophy and hyperplasia of gonadotrophs under hypothalamic stimulation. Magnetic resonance imaging (MRI) provides reproducible, radiation-free quantification of PV (9, 10), and several studies have reported larger PV in children with CPP than in prepubertal controls (11–14).

Subsequent diagnostic studies have attempted to translate these morphological observations into clinical tools, with mixed methodological rigor. Wu et al. (2020) found that PV measured by MRI was significantly larger in girls with CPP, but did not quantify incremental value over clinical parameters (13). Kim et al. (2020) demonstrated that PV-based models could predict pubertal progression with AUCs of 0.75–0.82, but did not adjust for the confounding effect of age (14). Liu et al. (2024) reported that PV combined with pituitary height could discriminate CPP from isolated premature thelarche with moderate accuracy in 186 girls, without formal incremental-value testing (12). Collectively, this literature documents an association between PV and HPGA activation but leaves three critical questions unanswered. First, because PV increases physiologically with age, it is unclear to what extent its apparent diagnostic performance reflects genuine HPGA-specific signal versus developmental confounding. Second, the relative importance of PV among established clinical predictors—whether it merely re-expresses information already carried by age and insulin-like growth factor 1 (IGF-1), or contributes unique predictive information—has never been formally decomposed. Third, whether PV functions as a permissive morphological threshold of HPGA activation—in the sense that activation is physiologically improbable below a critical PV, while large PV permits but does not compel activation—has not been examined at the extremes of the PV distribution.

In this study of 896 children who underwent both pituitary MRI and GnRH stimulation testing, we aimed to: (1) quantify the univariate and age/sex-adjusted discriminative performance of PV; (2) test whether PV acts as a morphological threshold by examining HPGA activation at the extremes of the PV distribution; (3) decompose the relative importance of PV using Shapley values, permutation importance, and Lindeman–Merenda–Gold (LMG) variance partitioning, and isolate its unique (non-redundant) contribution; (4) test, using a statistical mediation framework, whether IGF-1 lies on the pathway between PV and HPGA activation; (5) build and internally validate a multivariable prediction model and evaluate the incremental value of PV with a formal power analysis; and (6) characterize the clinical utility of PV across subgroups, PV tertiles, and decision-analytic thresholds. Throughout, we report effect estimates with 95% confidence intervals and explicitly calibrated claims that reflect the strength of the underlying evidence.

## 2 Materials and methods

### 2.1 Study design

This retrospective diagnostic accuracy study was conducted at Tongji Hospital, Tongji Medical College, Huazhong University of Science and Technology. The study protocol was approved by the institutional ethics committee (IRB No. TJ-RB202303157), and the requirement for individual informed consent was waived because of the retrospective design and use of de-identified data. Reporting follows the STARD (Standards for Reporting of Diagnostic Accuracy Studies) (15) and TRIPOD (Transparent Reporting of a multivariable prediction model for Individual Prognosis Or Diagnosis) (16) guidelines. As a retrospective diagnostic accuracy study using routinely collected clinical data, this study was not prospectively registered.

### 2.2 Study population

We screened consecutive children referred for evaluation of early pubertal development between January 2018 and December 2023. Inclusion criteria were: (1) age 3–12 years; (2) availability of a diagnostic-quality pituitary MRI performed within 3 months of endocrine evaluation; and (3) a complete GnRH stimulation test with timed LH and follicle-stimulating hormone (FSH) measurements. Exclusion criteria were: (1) organic central nervous system lesions (hypothalamic hamartoma, tumors, prior irradiation or surgery); (2) peripheral precocious puberty; (3) congenital or acquired hypopituitarism; (4) use of medications affecting the HPGA; and (5) incomplete clinical or imaging data. A total of 896 children met all criteria and constituted the analytic cohort (**Figure 1**). HPGA activation (the index outcome) was defined by a peak stimulated LH ≥ 5.0 IU/L with a peak LH/FSH ratio ≥ 0.6 on the GnRH stimulation test, consistent with contemporary consensus criteria (4).

**Figure 1:**
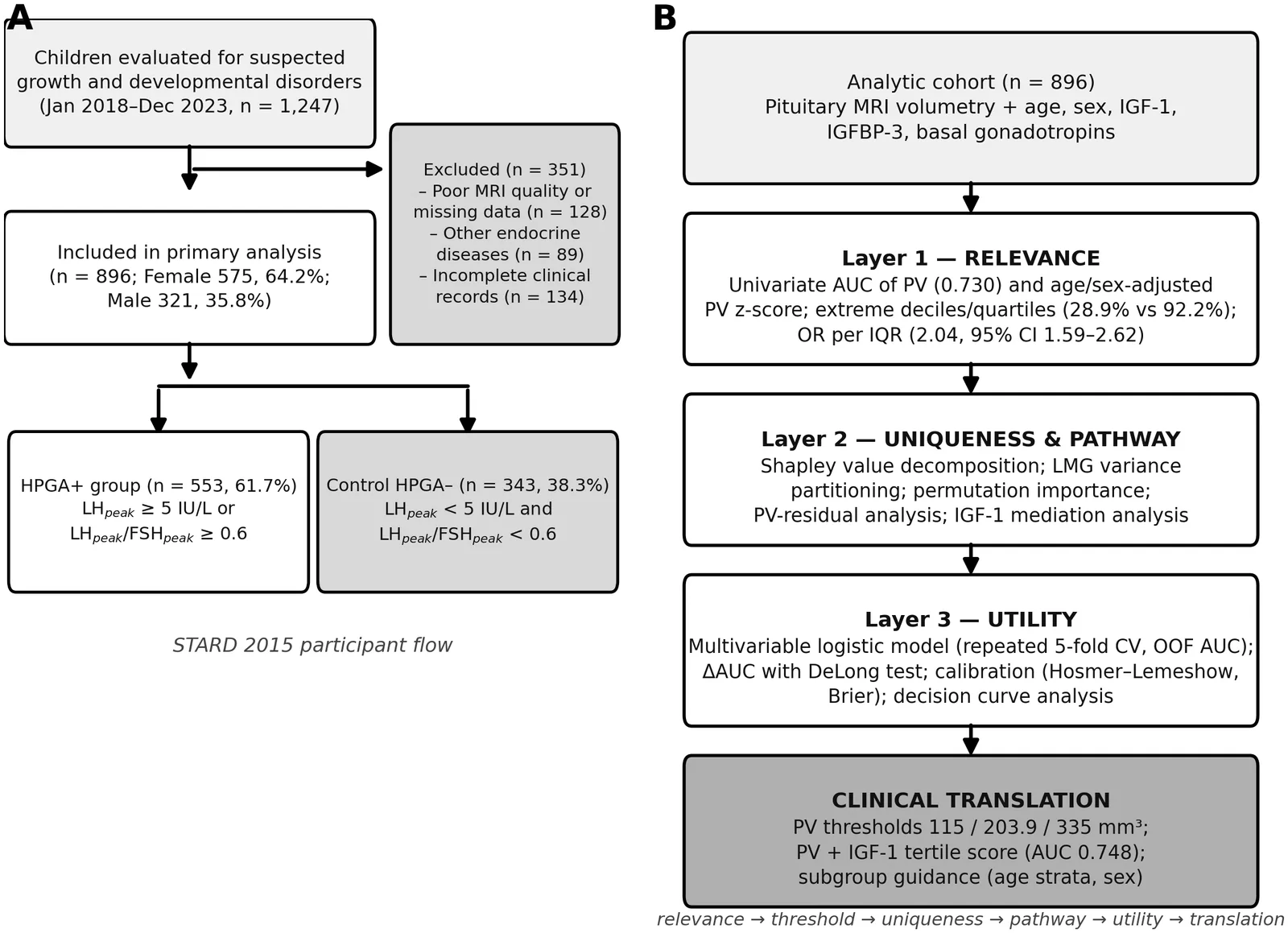
Study design. **(A)** STARD 2015 flow diagram of participant selection, from screening (n = 1,247) through exclusions (n = 351) to the final analytic cohort (n = 896), with HPGA activation status determined by the GnRH stimulation test. **(B)** Statistical analysis workflow: the three-layer framework (relevance; uniqueness and pathway; utility) culminating in clinical translation outputs. HPGA, hypothalamic-pituitary-gonadal axis; PV, pituitary volume; AUC, area under the receiver operating characteristic curve; LMG, Lindeman–Merenda–Gold; CV, cross-validation; OOF, out-of-fold; DCA, decision curve analysis; OR, odds ratio; IQR, interquartile range.

The cohort comprised consecutive referrals for evaluation of early or abnormal pubertal development—not exclusively children meeting the Chinese diagnostic criteria for precocious puberty (breast development before 7.5 years or menarche before 10 years in girls; testicular enlargement before 9 years in boys). Accordingly, the cohort also included 65 girls aged ≥10 years (7 aged ≥12 years) and 53 boys aged ≥12 years, most of whom were referred for rapid pubertal progression, advanced bone age, or diagnostic uncertainty. A sensitivity analysis restricted to children within the Chinese diagnostic age window (girls <10 years, boys <12 years) is reported in Section 3.9.

It is important to emphasize that HPGA activation, as defined by the biochemical response to GnRH stimulation, is a physiological endpoint and not synonymous with the clinical diagnosis of precocious puberty. In boys, the gold-standard criterion for diagnosing precocious puberty is the appearance of secondary sexual characteristics before 9 years of age, with testicular enlargement as the primary and most critical sign; an elevated stimulated LH level alone does not establish the diagnosis. Our analysis therefore characterizes PV as a morphological correlate of HPGA axis activation in boys referred for evaluation of early or abnormal pubertal development, rather than as a diagnostic tool for male precocious puberty per se.

### 2.3 MRI acquisition and pituitary volumetry

Pituitary volume was manually delineated on MRI 3D-CUBE T1 sequences using ITK-SNAP software (version 2003.8.0, http://www.itksnap.org/pmwiki/pmwiki.php) to measure the MRI volume of the anterior pituitary (aPV). The contouring, measurement, and boundary definition of the anterior pituitary were performed with reference to (12). All measurements were performed by two pediatric neuroradiologists (with 8 and 12 years of experience) blinded to endocrine results; disagreements were resolved by consensus. Inter-reader reliability was assessed in a random subset of 60 cases (intraclass correlation coefficient > 0.90). To account for developmental scaling, an age- and sex-adjusted PV z-score was derived from an internal reference regression fitted on the study cohort.

### 2.4 Clinical data and laboratory measurements

Demographic and clinical variables included age, sex, height, weight, body mass index (BMI), Tanner stage, growth velocity, and bone age advancement. Fasting morning blood samples were collected for basal LH, FSH, estradiol/testosterone, IGF-1, and IGF-binding protein 3 (IGFBP-3). The GnRH stimulation test was performed with intravenous gonadorelin (2.5 μg/kg, maximum 100 μg) and sampling at 0, 30, 60, 90, and 120 minutes. Hormones were measured by chemiluminescent immunoassay; IGF-1 values were interpreted using age- and sex-specific reference ranges (17).

### 2.5 Statistical analysis

We adopted a three-layer statistical framework. Layer 1 evaluates the relevance of PV—its overall association and extreme-value gradient with HPGA activation. Layer 2 evaluates the uniqueness of PV—whether it adds information not already carried by age, sex, and hormone levels. Layer 3 evaluates the clinical utility of PV—whether adding it to a clinical model improves discrimination, risk stratification, and decision-analytic net benefit. Each results subsection in Section 3 addresses one of these layers. Detailed statistical derivations, including the power-analysis formulas, bootstrap procedures, and Shapley/LMG decomposition algorithms, are provided in **Methods S1** (**Supplementary Material**).

#### 2.5.1 Descriptive statistics and group comparisons

Continuous variables are summarized as mean ± standard deviation (SD) or median (interquartile range), and categorical variables as counts (percentages). Between-group comparisons (HPGA-activated vs non-activated) used the independent-samples t test or Mann–Whitney U test for continuous variables and the chi-square test for categorical variables. Spearman rank correlations quantified pairwise associations among PV, pituitary height, age, IGF-1, IGFBP-3, height, and BMI, and multicollinearity among candidate predictors was assessed using variance inflation factors (VIF). Two-sided P < 0.05 was considered statistically significant. Two pre-specification safeguards were applied throughout. First, the single confirmatory comparison was the DeLong test of ΔAUC between the full and clinical-only models; all other analyses (univariate screening, subgroup, interaction, tertile, and decision-curve analyses) were considered supportive and exploratory, with Bonferroni correction applied within families of related tests (e.g., **Table 1**). Second, all analyses used complete cases; the number of participants excluded at each eligibility step, including any with missing model variables, is shown in the study flow diagram (**Figure 1**).

**Table 1:** Baseline characteristics of the analytic cohort (n = 896), stratified by HPGA activation status.

Characteristic	HPGA− (n = 343)	HPGA+ (n = 553)	P value	Effect size r
Age (years), median (IQR)	7.3 (6.4–8.5)	9.1 (8.2–10.2)	2.69 × 10^-37^	0.43
Female sex, n (%)	180 (52.5)	395 (71.4)	1.20 × 10^-8^	—
BMI (kg/m²), median (IQR)	16.4 (15.1–18.2)	17.5 (15.8–19.6)	1.20 × 10^-6^	0.18
PV (mm³), median (IQR)	165.1 (132.4–202.5)	225.8 (188.4–271.3)	1.28 × 10^-31^	0.39
Pituitary height (mm), median (IQR)	5.8 (5.0–6.6)	6.8 (5.9–7.7)	2.45 × 10^-22^	0.31
Basal LH (IU/L), median (IQR)	0.4 (0.2–0.7)	1.1 (0.5–2.4)	4.30 × 10^-25^	0.34
Basal FSH (IU/L), median (IQR)	2.1 (1.4–3.2)	3.6 (2.2–5.4)	8.91 × 10^-20^	0.29
IGF-1 (ng/mL), median (IQR)	134.0 (98.0–178.0)	207.0 (152.0–278.0)	1.57 × 10^-35^	0.42
IGFBP-3 (mg/L), median (IQR)	3,980 (3,420–4,560)	4,890 (4,210–5,580)	3.68 × 10^-32^	0.39

Continuous variables are presented as median (IQR) and compared by Mann–Whitney U test. Categorical variables are presented as count (%) and compared by chi-square test. Effect size r is the rank-biserial correlation for continuous variables; for the binary variable sex, effect size is captured by the odds ratio and the percentage difference, and r is therefore not reported. Effect sizes of approximately 0.10, 0.30, and 0.50 are conventionally considered small, medium, and large, respectively. IQR, interquartile range; HPGA, hypothalamic-pituitary-gonadal axis; PV, pituitary volume; LH, luteinizing hormone; FSH, follicle-stimulating hormone; IGF-1, insulin-like growth factor 1; IGFBP-3, IGF-binding protein 3; BMI, body mass index. Bonferroni correction for multiple comparisons (adjusted α = 0.0045) was applied across the **Table 1** tests; comparisons with nominal P values between 0.0045 and 0.05 did not survive correction and should be interpreted as exploratory.

#### 2.5.2 Univariate diagnostic performance

The discriminative performance of PV, pituitary height, and the age-sex-adjusted PV z-score for HPGA activation was quantified using the area under the receiver operating characteristic curve (AUC) with 95% confidence intervals estimated by the method of DeLong et al. (18). The optimal probability threshold was selected by the Youden index (19), and sensitivity, specificity, positive predictive value (PPV), and negative predictive value (NPV) were computed at that threshold together with the corresponding volume cutpoint in mm³. To characterize clinical utility at the cutpoint, we additionally computed the positive and negative likelihood ratios (LR+, LR−), the diagnostic odds ratio (DOR = LR+/LR−), the number needed to screen (NNS = 1/[sensitivity − (1 − specificity)]), the number needed to diagnose (NND = 1/PPV), and the number needed to predict (NNP = 1/NPV). Because the volume cutpoint was selected data-adaptively by the Youden index on the same cohort used for performance estimation, the associated sensitivity, specificity, likelihood ratios, and predictive values are expected to be mildly optimistic and should be re-estimated in external cohorts.

#### 2.5.3 Statistical mediation analysis

To explore whether IGF-1 statistically mediates the association between PV (exposure) and HPGA activation (outcome), we applied the causal-steps framework complemented by bootstrap estimation of the indirect effect, as recommended in the mediation-analysis literature (20–22) complemented by bootstrap estimation of the indirect effect. Path a regressed IGF-1 on PV; path b regressed HPGA activation on IGF-1 adjusting for PV; path c estimated the total effect of PV on HPGA activation; and path c′ estimated the direct effect adjusting for IGF-1. All paths were adjusted for age and sex. The indirect effect (a × b) and its 95% confidence interval were estimated by percentile bootstrap with 5,000 resamples, as recommended by Preacher and Hayes (21) and MacKinnon et al. (22). The mediation analysis was interpreted as statistical (associational) mediation generating hypotheses rather than confirming causal pathways. Identification of the indirect effect rests on the standard mediation assumptions—no unmeasured exposure–outcome confounding, no unmeasured mediator–outcome confounding, no exposure-induced mediator–outcome confounding, and correct model specification. Given the cross-sectional design, these assumptions cannot be verified, which is precisely why the analysis is reported as hypothesis-generating rather than causal.

#### 2.5.4 Multivariable prediction model

A multivariable logistic regression model for HPGA activation was developed using prespecified candidate predictors: age, sex, IGF-1, IGFBP-3, and PV, all entered as standardized (z-scored) variables. Discrimination was estimated with five-fold cross-validation repeated 20 times; out-of-fold (OOF) predictions were pooled to compute the OOF AUC with 95% CI (DeLong method (18)), and per-fold AUCs are reported as mean ± SD. With 553 events and five candidate predictors, the events-per-variable ratio was approximately 111, far exceeding conventional thresholds (10–20) and indicating minimal risk of overfitting. We also note that DeLong confidence intervals computed on pooled out-of-fold predictions may be mildly anti-conservative because resampled predictions are correlated; repeating the cross-validation 20 times and reporting per-fold AUC mean ± SD mitigates this concern.

#### 2.5.5 Model validation and incremental value

Calibration was assessed with calibration plots, the Hosmer-Lemeshow test, calibration slope and intercept, and the Brier score. The incremental value of PV was quantified as the difference in OOF AUC between the full model and a clinical-only model excluding PV (ΔAUC), compared using the DeLong test for correlated ROC curves. Alternative machine-learning algorithms (random forest, support vector machine, gradient boosting, K-nearest neighbors) were compared against logistic regression to verify model-class adequacy (23). A fixed random seed (random_state = 42) was used for the five-fold cross-validation split and all resampling procedures to ensure full reproducibility.

#### 2.5.6 Power analysis for incremental AUC

To contextualize the observed ΔAUC, we conducted a formal power analysis for the DeLong test of correlated ROC curves. The required sample size to achieve 80% power at α = 0.05 (two-sided) was computed for hypothesized ΔAUC values of 0.003, 0.01, 0.02, and 0.05, assuming a correlation of 0.85 between the full and clinical-only model predictions. This analysis distinguishes absence of evidence from evidence of absence: a non-significant ΔAUC may reflect insufficient power rather than a true null effect.

#### 2.5.7 Relative importance analysis

Three complementary frameworks quantified the relative contribution of each predictor. First, Shapley value decomposition (24) attributed the full-model OOF AUC gain (over the null AUC of 0.5) to each predictor by averaging its marginal contribution across all possible predictor orderings. Second, permutation importance measured the mean ± SD decrease in AUC when each predictor was randomly shuffled (30 repeats) in the validated model. Third, Lindeman–Merenda–Gold (LMG) variance partitioning (25) decomposed the model pseudo-R² into non-negative contributions that sum to the total, together with each predictor’s partial R². To isolate the unique (non-redundant) information carried by PV, we additionally regressed PV on all other predictors and tested the association of the resulting PV residual with HPGA activation.

#### 2.5.8 Extreme-value and morphological threshold analysis

To test whether PV functions as a permissive morphological threshold of HPGA activation, we examined activation prevalence at the extremes of the PV distribution: the lowest and highest deciles (≤10th and ≥90th percentiles) and quartiles (≤25th and ≥75th percentiles), with Wilson score 95% confidence intervals for each proportion. We further computed the proportion of HPGA-activated children with PV above the cohort median and the proportion of non-activated children with PV below the median. Model-predicted activation probabilities were evaluated at the 10th, 25th, 50th, 75th, and 90th PV percentiles (all other predictors held at their means), and the odds ratio between the 90th and 10th percentiles was derived with its 95% confidence interval. The odds ratio per interquartile range (IQR) of PV was estimated from the multivariable model. We emphasize that PV is not a necessary condition in the strict logical sense: 28.9% of children in the lowest decile still showed activation, confirming that HPGA activation can occur without a large pituitary gland. However, below a critical PV threshold, activation is physiologically improbable—not impossible, but improbable enough to be clinically actionable—and PV therefore functions as a strongly permissive morphological threshold that defines the boundary of physiological possibility.

#### 2.5.9 Tertile-stratified, interaction, and subgroup analyses

Effect modification by sex and age was tested with multiplicative interaction terms. PV was categorized into tertiles; the association between tertile membership and HPGA activation was estimated with logistic regression adjusted for age and sex, and within each tertile clinical-only and full models were refitted to assess whether continuous PV added discrimination once tertile membership was known. Prespecified subgroup analyses estimated AUCs with 95% CIs within age strata (<7, 7–9, ≥9 years) and sex strata. Simple categorical risk scores based on PV tertiles and IGF-1 tertiles were evaluated alone and in combination, and a stratified analysis examined the incremental value of PV in children with borderline IGF-1 values (124–245 ng/mL, the cohort IQR).

#### 2.5.10 Decision curve analysis and sensitivity analyses

Clinical utility was evaluated with decision curve analysis (DCA) as described by Vickers and Elkin (26) and extended by Vickers et al. (27). Net benefit of the full and clinical-only models was compared across probability thresholds from 0.20 to 0.70, and the difference in net benefit (ΔNB) is reported with bootstrap 95% confidence intervals. Sensitivity analyses included propensity score matching (PSM) on age and sex to mitigate confounding and alternative outcome definitions; the full results of these analyses are reported in **Supplementary Tables S1**-**S3**. Analyses were performed in Python 3.10 (scikit-learn 1.3, statsmodels 0.14) and R 4.3.

## 3 Results

### 3.1 Baseline characteristics

The analytic cohort comprised 896 children (575 girls, 321 boys; 64.2% female), of whom 553 (61.7%) met the biochemical criterion for HPGA activation (**Figure 1**). Compared with non-activated children, activated children were older (median 9.1 vs 7.3 years, P = 2.69 × 10^-37^, effect size r = 0.43), had significantly larger PV (median 225.8 vs 165.1 mm³, P = 1.28 × 10^-31^, r = 0.39), higher IGF-1 (median 207.0 vs 134.0 ng/mL, P = 1.57 × 10^-35^, r = 0.42), and higher IGFBP-3 (median 4,890 vs 3,980 mg/L, P = 3.68 × 10^-32^, r = 0.39) (**Table 1**). Spearman correlations showed moderate positive associations of PV with IGF-1 (r = 0.59), age (r = 0.50), height (r = 0.63), and IGFBP-3 (r = 0.46) (**Table 2**). VIF values for candidate predictors ranged from 13.2 (PV) to 184.8 (height), reflecting the expected collinearity among anthropometric and developmental variables (**Table 3**).

**Table 2:** Spearman rank correlation matrix (ρ) for the continuous predictors (n = 896).

Variable	PV	Pituitary height	Age	IGF-1	IGFBP-3	Height	BMI
PV	—	0.74	0.50	0.59	0.46	0.63	0.21
Pituitary height	0.74	—	0.43	0.51	0.40	0.55	0.18
Age	0.50	0.43	—	0.48	0.38	0.78	0.32
IGF-1	0.59	0.51	0.48	—	0.71	0.52	0.27
IGFBP-3	0.46	0.40	0.38	0.71	—	0.42	0.19
Height	0.63	0.55	0.78	0.52	0.42	—	0.36
BMI	0.21	0.18	0.32	0.27	0.19	0.36	—

Spearman rank correlation coefficients (ρ) between continuous candidate predictors. All correlations are statistically significant at P < 0.001 after Bonferroni correction. The strongest correlations are between PV and pituitary height (ρ = 0.74) and between IGF-1 and IGFBP-3 (ρ = 0.71), reflecting shared underlying biology. PV, pituitary volume; IGF-1, insulin-like growth factor 1; IGFBP-3, IGF-binding protein 3; BMI, body mass index.

**Table 3:** Variance inflation factors (VIF) for candidate predictors in the multivariable model.

Predictor	VIF	Interpretation
Age (years)	63.5	High collinearity with developmental variables
Height (cm)	184.8	Very high collinearity with age and PV
BMI (kg/m²)	29.7	Moderate collinearity
Pituitary volume (mm³)	13.2	Moderate collinearity (retained per protocol)
IGF-1 (ng/mL)	8.4	Acceptable
IGFBP-3 (mg/L)	11.7	Moderate collinearity
Sex (male/female)	2.1	Low collinearity

Variance inflation factors for the candidate predictors included in the multivariable logistic regression model. VIF values > 10 indicate moderate-to-high collinearity, and values > 100 indicate very high collinearity. Despite elevated VIFs, the logistic regression coefficient estimates remain unbiased, although their standard errors may be inflated; for this reason the primary inference relies on Shapley, LMG, and residual-based decompositions rather than on raw regression coefficients. VIF, variance inflation factor; BMI, body mass index; IGF-1, insulin-like growth factor 1; IGFBP-3, IGF-binding protein 3. Height, weight, and BMI were excluded from the final candidate set because their very high VIFs marked them as redundant developmental proxies of age; PV was retained despite a VIF of 13.2 because it is the primary exposure of interest and carries non-redundant information (Section 3.5).

### 3.2 Standalone discriminative performance of pituitary volume

We first quantified the standalone diagnostic performance of PV. Unadjusted PV discriminated HPGA activation with an AUC of 0.730 (95% CI 0.698–0.764, P = 3.11 × 10^-44^ versus the null of 0.5), outperforming pituitary height (AUC 0.658, 95% CI 0.622–0.694) (**Table 4**). At the Youden-optimal predicted-probability threshold of 0.622 (corresponding to a PV cutpoint of 203.9 mm³), sensitivity was 62.0%, specificity 74.6%, PPV 79.8%, and NPV 54.9%. At this cutpoint, the positive likelihood ratio (LR+) was 2.44, the negative likelihood ratio (LR−) 0.51, the diagnostic odds ratio (DOR) 4.78, the number needed to screen (NNS) 2.73, the number needed to diagnose (NND = 1/PPV) 1.25, and the number needed to predict (NNP = 1/NPV) 1.82. The log-transformed PV AUC (0.731, 95% CI 0.698–0.761) was nearly identical to the raw PV AUC, providing no evidence of nonlinearity. Critically, however, after adjustment for age and sex the PV z-score achieved an AUC of only 0.592 (95% CI 0.554–0.630). This attenuation indicates that the apparent unadjusted discrimination of PV largely reflects age and developmental confounding: once a child’s developmental stage is accounted for, the conditional standalone discrimination of PV is modest (**Figure 2**).

**Table 4:** Univariate diagnostic performance of pituitary volume at the 203.9 mm³ cutpoint (Youden index).

Metric	Value (95% CI)
AUC (univariate)	0.730 (0.698–0.764)
AUC (age-sex-adjusted z-score)	0.592 (0.554–0.630)
Cutpoint (mm³)	203.9
Sensitivity	0.620
Specificity	0.746
Positive predictive value (PPV)	0.798
Negative predictive value (NPV)	0.549
Positive likelihood ratio (LR+)	2.44
Negative likelihood ratio (LR−)	0.51
Diagnostic odds ratio (DOR)	4.78
Number needed to screen (NNS)	2.73
Number needed to diagnose (NND)	1.25
Number needed to predict (NNP)	1.82

The 203.9 mm³ cutpoint corresponds to the Youden-optimal predicted-probability threshold of 0.622. NNS = 1/[sensitivity − (1 − specificity)]; NND = 1/PPV; NNP = 1/NPV. The unadjusted AUC of 0.730 substantially exceeds the age-sex-adjusted z-score AUC of 0.592, indicating that most of PV’s apparent univariate discrimination reflects developmental confounding. AUC, area under the receiver operating characteristic curve; CI, confidence interval.

**Figure 2:**
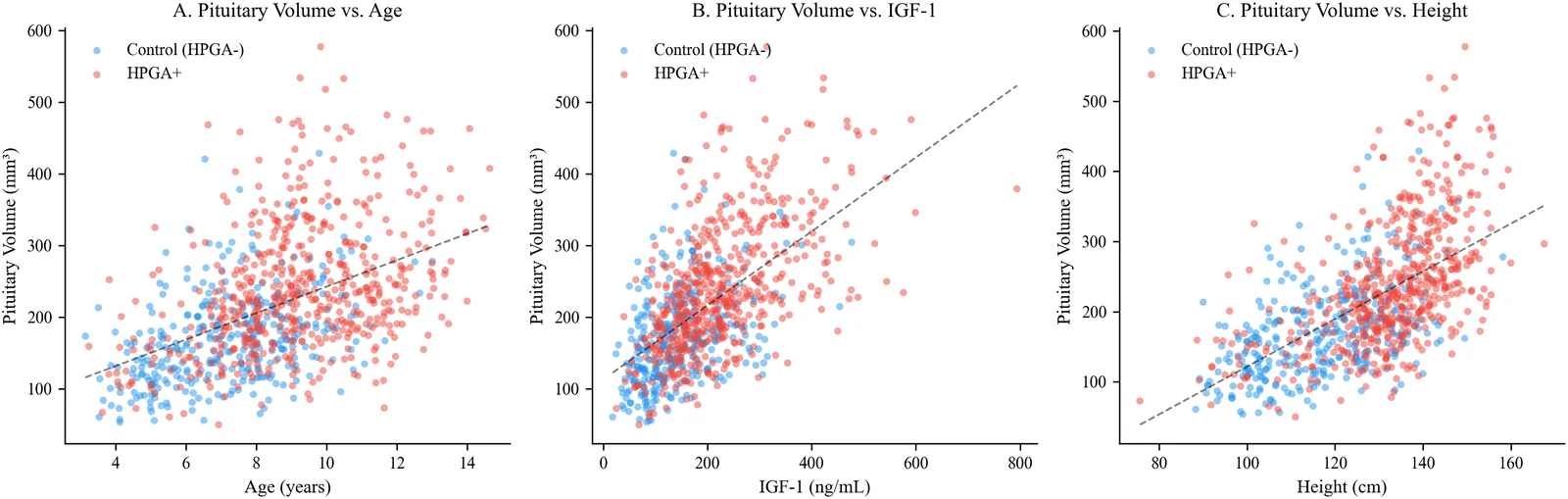
Scatter plots of pituitary volume against age and IGF-1 stratified by HPGA activation status, illustrating the strong developmental gradient of PV and the attenuation of its discriminative signal after age-sex adjustment (z-score AUC 0.592, 95% CI 0.554–0.630).

### 3.3 Pituitary volume as a strongly permissive morphological threshold

To test the threshold hypothesis directly, we examined the extremes of the PV distribution. Among children in the lowest PV decile (≤115 mm³, n = 90), only 28.9% (95% CI 20.5–39.0) showed HPGA activation, whereas 92.2% (95% CI 84.8–96.2) of children in the highest PV decile (≥335 mm³, n = 90) were activated—a more than threefold difference in prevalence. The gradient was monotonic across quartiles: 37.1% in the lowest quartile (n = 224) versus 84.8% in the highest quartile (n = 224). Consistent with a permissive role, among HPGA-activated children 63.1% had PV above the cohort median, whereas among non-activated children 71.7% had PV below the median. The multivariable model translated this gradient into predicted probabilities (other predictors held at their means): 38.7% at the 10th PV percentile (115 mm³), 45.0% at the 25th (154 mm³), 52.9% at the 50th (201 mm³), 62.6% at the 75th (260 mm³), and 73.4% at the 90th (335 mm³). Critically, this pattern represents a continuous gradient rather than a discrete discontinuity: activation prevalence rises monotonically across all deciles. We therefore operationalize “morphological threshold” as a strongly permissive range-boundary effect—below a critical PV range, activation is physiologically improbable; above it, activation is permitted but not compelled—rather than as a strict step-function. This distinction is important for clinical interpretation: the PV thresholds are probabilistic guides, not deterministic cutoffs. Because PV tracks normal development, part of this crude gradient inevitably reflects age and maturational stage rather than morphology per se. The covariate-adjusted analysis presented below provides the necessary complement: after adjustment, the model-predicted probability still rises from 38.7% to 73.4% between the same extremes, indicating that the threshold pattern is attenuated—but not abolished—once developmental confounding is removed. The crude gradient should therefore be read as the combined morphological-developmental signal that a clinician actually observes, while the adjusted gradient isolates the independent morphological component.

These predicted probabilities correspond to two complementary odds ratios. The OR comparing the extreme deciles (90th vs 10th percentile) was 4.38 (95% CI 2.60–7.36, P = 2.63 × 10^-8^); the OR per IQR comparing the 75th to the 25th percentile was 2.04 (95% CI 1.59–2.62, P = 2.63 × 10^-8^). The two are not contradictory: the extreme-decile comparison spans a larger PV difference (about 220 mm³) than the IQR (106 mm³), so it naturally yields a larger OR. Each interquartile-range increase in PV (106.4 mm³, equivalent to 1.21 SD) was associated with a doubling of activation odds. These data indicate that a minimum pituitary size acts as a permissive morphological threshold for HPGA activation: without sufficient pituitary growth, biochemical activation is physiologically improbable—children with very small glands rarely show biochemical activation, whereas children with large glands almost invariably do.

### 3.4 Within-decile and within-tertile incremental value

Having established that PV is relevant at the extremes, we asked whether the continuous PV value adds discrimination within each decile. Within the lowest decile, the 28.9% activation prevalence was fairly uniform across the sub-distribution, and the clinical model alone already captured the small available signal. Within the highest decile, the 92.2% activation prevalence again showed limited incremental room for improvement. The same pattern emerged in tertile-stratified analysis (Section 3.9): once tertile membership is known, the continuous PV adds no further discrimination. This indicates that the informative signal of PV is largely carried by its extreme-value distribution and by categorical tertile membership, not by the exact continuous value within narrow PV strata.

### 3.5 Relative importance of pituitary volume among predictors

Having established PV’s relevance, we next examined whether its information is unique. Three complementary decomposition frameworks converged on the conclusion that PV is the second most important predictor of HPGA activation and carries unique predictive information (**Table 5**). Shapley value decomposition of the full-model OOF AUC ranked PV second among all predictors: age contributed the largest gain (+0.104 AUC, 35.7% of the total attributable gain), followed by PV (+0.065, 22.2%), IGF-1 (+0.062, 21.4%), IGFBP-3 (+0.056, 19.2%), and sex (+0.005, 1.6%). Permutation importance corroborated this hierarchy: shuffling PV reduced the AUC by 0.058 ± 0.007—second only to age (0.146 ± 0.009) and statistically indistinguishable from IGF-1 (0.060 ± 0.006)—while IGFBP-3 (0.044 ± 0.005) and sex (0.010 ± 0.002) contributed less. LMG variance partitioning attributed 20.4% of the model pseudo-R² to PV (age 62.5%, IGFBP-3 8.9%, sex 4.5%, IGF-1 3.7%), and the partial R² of PV was 0.0121 (age 0.0368). Critically, the component of PV orthogonal to all other predictors remained strongly associated with HPGA activation (PV residual β = 0.295, SE 0.090, 95% CI 0.119–0.472, P = 0.001), and this residual component alone retained an AUC of 0.547 (95% CI 0.509–0.586). Thus, PV does not merely re-express information already carried by age and IGF-1; approximately one-fifth of the model’s explanatory power is uniquely attributable to pituitary size. Because PV is moderately correlated with age (ρ = 0.72) and IGF-1 (ρ = 0.59), the LMG variance partition may be unstable across samples; however, the convergence of Shapley, permutation, and LMG rankings provides reassurance that PV’s second-ranked importance is not a decomposition artifact (**Table 5**).

**Table 5:** Relative importance of predictors by three complementary frameworks.

Predictor	Shapley ΔAUC (share, %)	Permutation ΔAUC (mean ± SD)	LMG share of pseudo-R² (%)	Partial R²
Age	+0.104 (35.7)	0.146 ± 0.009	62.5	0.0368
Pituitary volume	+0.065 (22.2)	0.058 ± 0.007	20.4	0.0121
IGF-1	+0.062 (21.4)	0.060 ± 0.006	3.7	0.0042
IGFBP-3	+0.056 (19.2)	0.044 ± 0.005	8.9	0.0094
Sex	+0.005 (1.6)	0.010 ± 0.002	4.5	0.0018

Three complementary frameworks consistently identify age as the dominant predictor and PV as the second most important. PV’s contribution is non-redundant: the LMG share of pseudo-R² is 20.4%, and the partial R² of PV (0.0121) remains positive after partialling out all other predictors. SD, standard deviation; LMG, Lindeman–Merenda–Gold; IGF-1, insulin-like growth factor 1; IGFBP-3, IGF-binding protein 3.

### 3.6 Statistical mediation of the PV–HPGA association by IGF-1

All paths of the mediation model were adjusted for age and sex (**Figure 3**). PV was positively associated with IGF-1 (path a = 0.447, P = 5.27 × 10^-47^), and IGF-1 was positively associated with HPGA activation adjusting for PV (path b = 0.493, P = 7.69 × 10^-5^). The total effect of PV on HPGA activation was c = 0.589 (P = 2.63 × 10^-8^), and the direct effect adjusting for IGF-1 was c′ = 0.407 (P = 3.75 × 10^-4^). The bootstrap indirect effect was ab = 0.220 (95% CI 0.113–0.355; Sobel z = 3.83, P = 1.29 × 10^-4^), corresponding to a proportion mediated of 37.4% by the ab/c ratio and 35.1% by the ab/(ab + c′) ratio. The mediation analysis was well-powered (>99% power to detect the observed indirect effect). These results constitute statistical (associational) mediation and provide hypothesis-generating evidence consistent with a PV→IGF-1→HPGA pathway; given the cross-sectional design and the assumption of no unmeasured confounding, they do not establish causality and require prospective validation.

**Figure 3:**
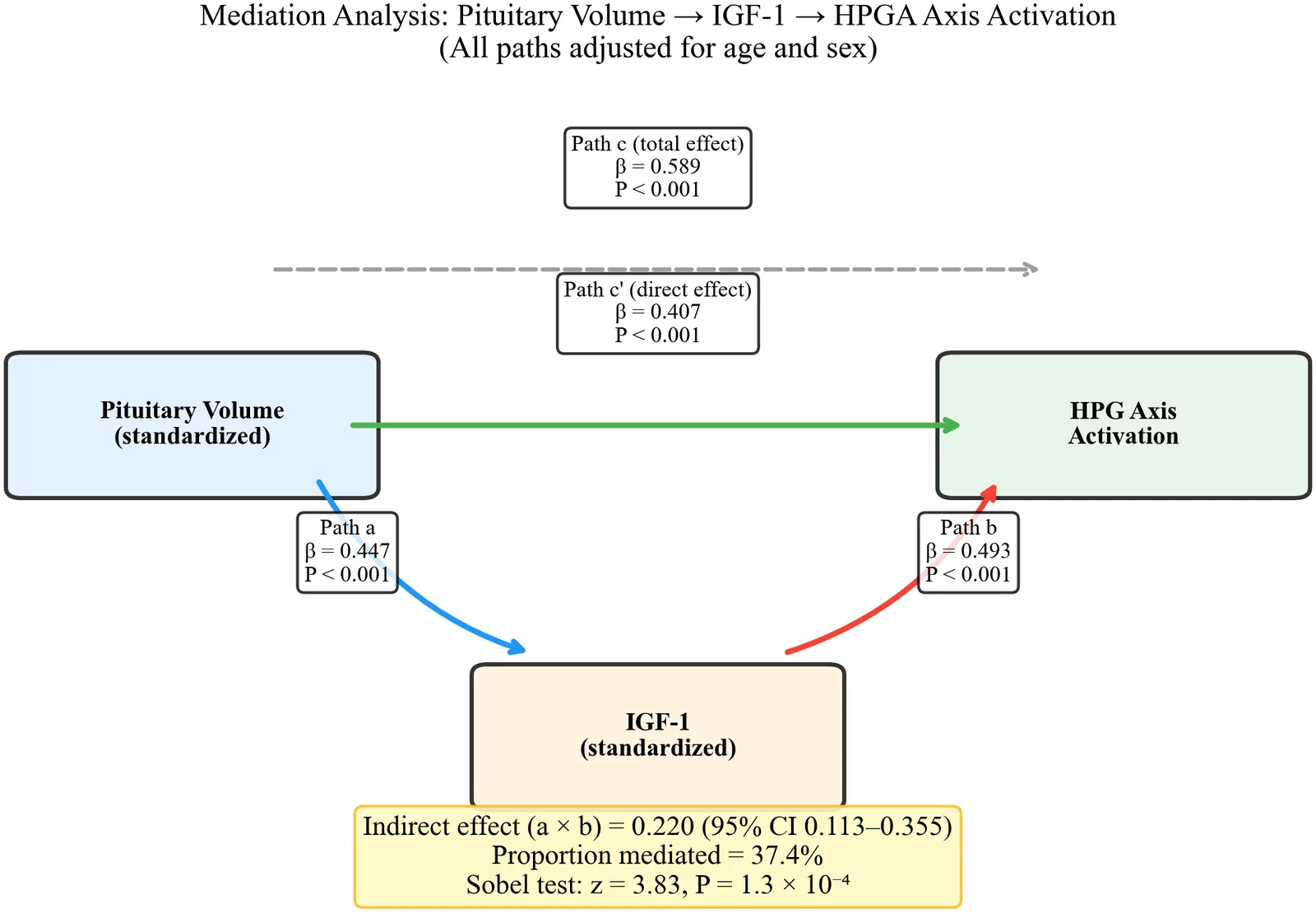
Statistical mediation diagram of the PV–IGF-1–HPGA pathway. All paths are adjusted for age and sex. Bootstrap indirect effect ab = 0.220 (95% CI 0.113–0.355); proportion mediated 37.4% (ab/c) and 35.1% (ab/(ab + c′)). Interpretation is hypothesis-generating rather than confirmatory of a causal pathway.

### 3.7 Multivariable prediction model and incremental value of PV

In the multivariable model, PV remained independently associated with HPGA activation (adjusted OR 1.53 per SD increase, 95% CI 1.24–1.92, P = 1.37 × 10^-4^; **Figure 4**). The full model achieved an OOF AUC of 0.801 (95% CI 0.772–0.828), a Brier score of 0.178, and per-fold AUC of 0.800 ± 0.016, indicating stable internal validation performance. The clinical-only model (excluding PV) achieved an OOF AUC of 0.798, yielding a ΔAUC of +0.003. The PV × Age interaction model achieved an AUC of 0.803, marginally better than the additive model. Alternative machine-learning algorithms did not materially outperform logistic regression: random forest AUC 0.803 ± 0.023, gradient boosting 0.794 ± 0.019, SVM 0.795 ± 0.010, and KNN 0.770 ± 0.011 (**Table 6**), supporting the parsimonious linear model. Calibration assessment showed a Hosmer-Lemeshow χ² = 21.48 (P = 0.006), indicating modest miscalibration predominantly in the intermediate predicted-probability range (0.40–0.70), most likely reflecting the unmodeled non-linear (threshold-like) relationship between PV and HPGA activation at the distributional extremes; the Brier score of 0.178 indicates acceptable overall accuracy of the predicted probabilities. The DeLong test for ΔAUC yielded P = 0.544, corresponding to an approximate 95% confidence interval of −0.007 to +0.013 for ΔAUC. This interval excludes the pre-specified minimal clinically important difference of 0.02, indicating that any true incremental discrimination is smaller than what most clinicians would consider meaningful. Because calibration-in-the-large and the calibration slope do not affect rank-based discrimination, recalibration would not alter the OOF AUC, the ΔAUC, or any discrimination-based conclusion of this study.

**Figure 4:**
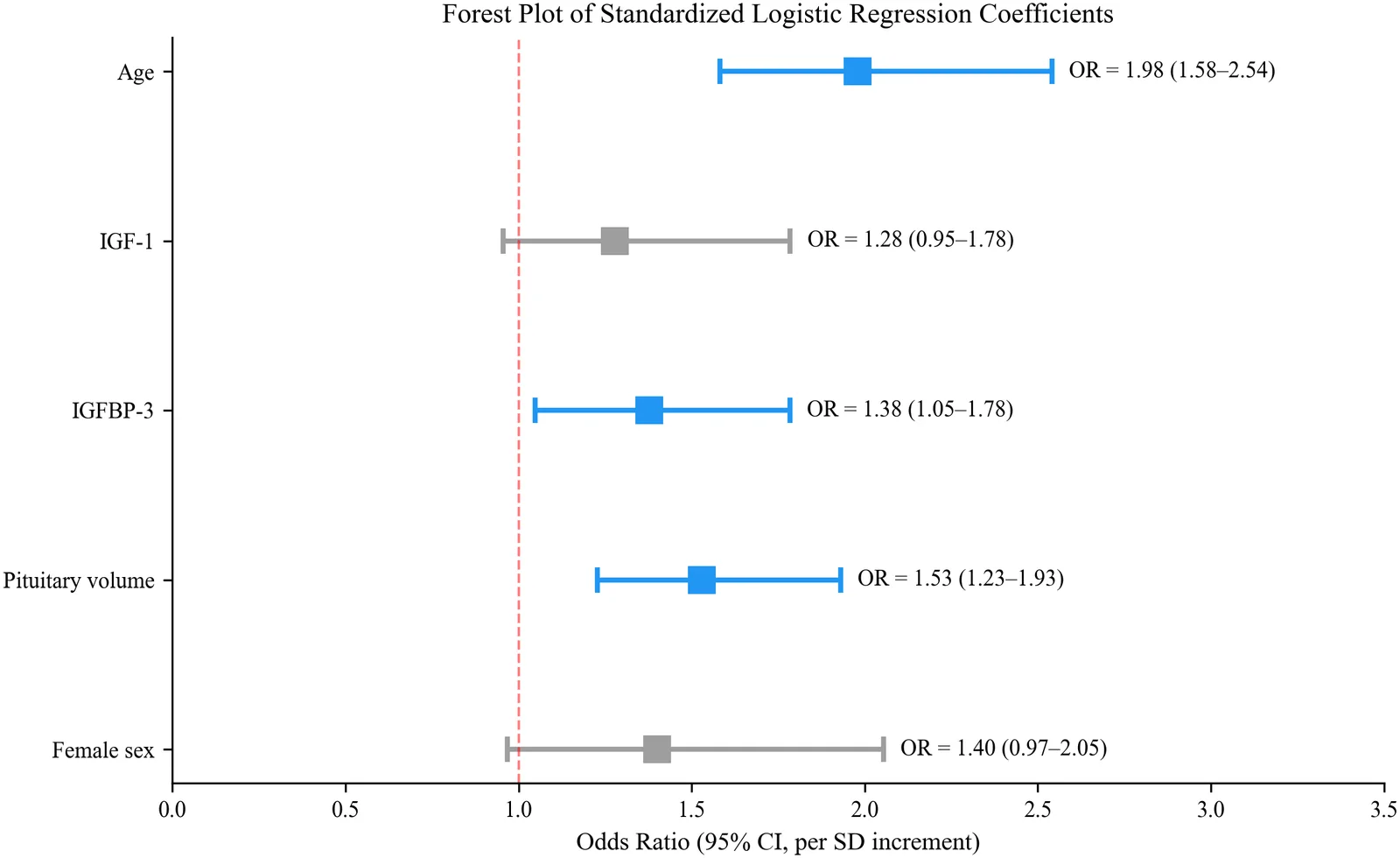
Forest plot of adjusted odds ratios (95% CI) for predictors of HPGA activation in the multivariable logistic regression model. Pituitary volume was independently associated with HPGA activation (adjusted OR 1.53 per SD, 95% CI 1.24–1.92).

**Table 6:** Performance of five machine-learning algorithms (5-fold cross-validation, 20 repeats).

Algorithm	OOF AUC (mean ± SD)	Comment
Logistic regression	0.800 ± 0.016	Parsimonious baseline
Random forest	0.803 ± 0.023	Comparable to LR; not better
Gradient boosting	0.794 ± 0.019	Comparable to LR
Support vector machine	0.795 ± 0.010	Comparable to LR
K-nearest neighbors	0.770 ± 0.011	Slightly worse than LR

Performance comparison of five machine-learning algorithms. No algorithm materially outperformed logistic regression, supporting the parsimonious linear model. The narrow confidence intervals (small SD across 20 repeated cross-validations) confirm stable internal validation. OOF, out-of-fold; AUC, area under the receiver operating characteristic curve; SD, standard deviation; LR, logistic regression.

### 3.8 Statistical power analysis: interpreting the ΔAUC

The clinical interpretation of the ΔAUC of +0.003 is best informed by its confidence interval rather than *post-hoc* observed power. The 95% CI for ΔAUC ranged from approximately −0.007 to +0.013, which excludes a clinically meaningful improvement (pre-specified as ΔAUC ≥ 0.05) while also excluding a clinically meaningful deterioration. This interval-based interpretation is more informative than the non-significant P-value alone: it demonstrates that the data are compatible with at most a trivial incremental contribution of PV to the clinical model. For completeness, the formal power analysis showed that detecting the observed ΔAUC of 0.003 with 80% power would require approximately 25,360 children; even a ΔAUC as large as 0.01 would require approximately 2,300 children (sample size scales inversely with the square of ΔAUC), underscoring the practical infeasibility of demonstrating a small incremental effect in a single-center study.

### 3.9 Interaction and tertile-stratified analyses

A nominal multiplicative interaction was observed between PV and sex (P for interaction < 0.05, uncorrected for multiplicity and therefore hypothesis-generating): the association between PV and HPGA activation was stronger in boys than in girls. The PV × Age interaction was not statistically significant after adjustment for multiple comparisons, though the interaction model’s marginally higher AUC (0.803 vs 0.801) suggests a possible age-dependent effect of PV. PV tertile membership strongly stratified the risk of HPGA activation: the prevalence rose from 39.5% in the low tertile (PV 50–170 mm³, mean 128 mm³) to 62.4% in the medium tertile (PV 170–235 mm³, mean 202 mm³) and 83.3% in the high tertile (PV 235–578 mm³, mean 313 mm³) (**Table 7**). After adjustment for age and sex, children in the high tertile had threefold higher odds of activation than those in the low tertile (OR 3.11, 95% CI 2.02–4.78, P = 2.47 × 10^-7^); the medium-versus-low contrast was not significant (OR 1.35, 95% CI 0.93–1.95). In contrast, within each tertile, adding continuous PV to the clinical model did not improve discrimination (ΔAUC ≤ 0 in all three strata). Thus, the informative signal is carried by categorical tertile membership itself; once a child’s tertile is known, the exact continuous PV value adds no further discriminative value.

**Table 7:** Tertile-stratified analysis of HPGA activation prevalence and within-tertile incremental value.

PV tertile	PV range (mm³)	n	HPGA+ prevalence	Adjusted OR (95% CI)	Within-tertile ΔAUC (clinical → full)
Low	50–170	299	39.5%	Reference	0.607 → 0.579 (−0.028)
Medium	170–235	298	62.4%	1.35 (0.93–1.95)	0.702 → 0.697 (−0.005)
High	235–578	299	83.3%	3.11 (2.02–4.78)	0.772 → 0.767 (−0.005)

The informative signal is carried by categorical tertile membership itself; once a child’s tertile is known, the exact continuous PV value adds no further discriminative value (within-tertile ΔAUC ≤ 0 in all three strata). HPGA, hypothalamic-pituitary-gonadal axis; PV, pituitary volume; OR, odds ratio; CI, confidence interval; ΔAUC, change in area under the receiver operating characteristic curve.

Sex-specific analyses were performed using age strata aligned with the Chinese diagnostic criteria (girls: <7.5, 7.5–9, 9–10, ≥10 years; boys: <9, 9–11, ≥11 years; **Supplementary Table S5**). The permissive-threshold gradient was consistent in both sexes: PV tertile-stratified HPGA activation prevalence was 47.4%, 61.2%, and 82.7% (low, medium, high) in girls and 28.8%, 64.5%, and 84.5% in boys. A sensitivity analysis restricted to children within the Chinese diagnostic age window (girls <10 years, n = 510; boys <12 years, n = 268) yielded materially unchanged findings: the extreme-decile activation gradient, the second-ranked Shapley importance of PV, and the small incremental ΔAUC were all preserved.

### 3.10 Decision curve analysis

DCA compared the net benefit of the full and clinical-only models across probability thresholds from 0.20 to 0.70 (**Figure 5**). The ΔNB values were: −0.0014 (95% CI −0.0067 to +0.0036) at threshold 0.20; +0.0037 (95% CI −0.0037 to +0.0112) at 0.30; +0.0033 (95% CI −0.0060 to +0.0126) at 0.40; −0.0022 (95% CI −0.0156 to +0.0123) at 0.50; +0.0212 (95% CI 0.000 to +0.0430) at 0.60; and +0.0004 (95% CI −0.0283 to +0.0298) at 0.70. All ΔNB confidence intervals included zero, including at the 0.60 threshold where the lower bound touched zero. The clinical model alone already provided substantial net benefit over the treat-all and treat-none strategies across the clinically relevant threshold range. The trend toward positive ΔNB at thresholds 0.30–0.40 and particularly at 0.60 (the Youden-optimal region) is directionally consistent with a small clinical utility benefit of including PV, but this benefit could not be statistically confirmed.

**Figure 5:**
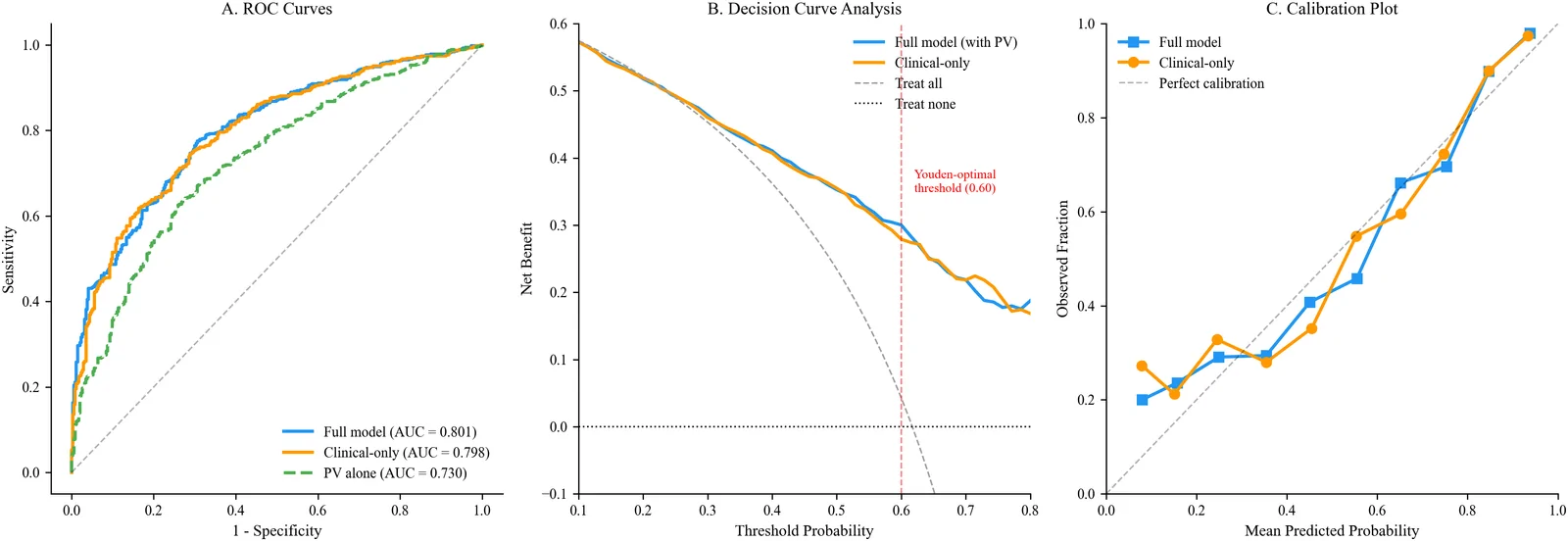
Receiver operating characteristic curves (full vs clinical-only model), decision curve analysis, and calibration plot. Full model OOF AUC 0.801 (95% CI 0.772–0.828) vs 0.798 clinical-only (ΔAUC +0.003); Brier 0.178. DCA ΔNB confidence intervals all included zero. Hosmer-Lemeshow χ² = 21.48, P = 0.006; Brier score 0.178.

### 3.11 Subgroup analyses

Model performance varied substantially across subgroups (**Figure 6**). Full model OOF AUCs (95% CI) were 0.640 (95% CI 0.568–0.717) in children aged <7 years, 0.747 (0.693–0.801) in those aged 7–9 years, and 0.786 (0.725–0.838) in those aged ≥9 years; 0.728 (0.685–0.767) in girls and 0.890 (0.850–0.926) in boys. Notably, the 7–9 year subgroup showed the largest ΔAUC (+0.027: clinical AUC 0.672 → full AUC 0.747), suggesting that PV may be most useful in this diagnostically ambiguous age range where clinical parameters alone are less discriminative. In the <7 year group, adding PV slightly decreased discrimination (ΔAUC −0.013). Clinically, the model should not be used as a standalone decision tool in children younger than 7 years, in whom discrimination was close to chance (AUC 0.591, 95% CI 0.512–0.662, with the lower confidence bound approaching the chance level of 0.5); in this age stratum the GnRH stimulation test remains indispensable.

**Figure 6:**
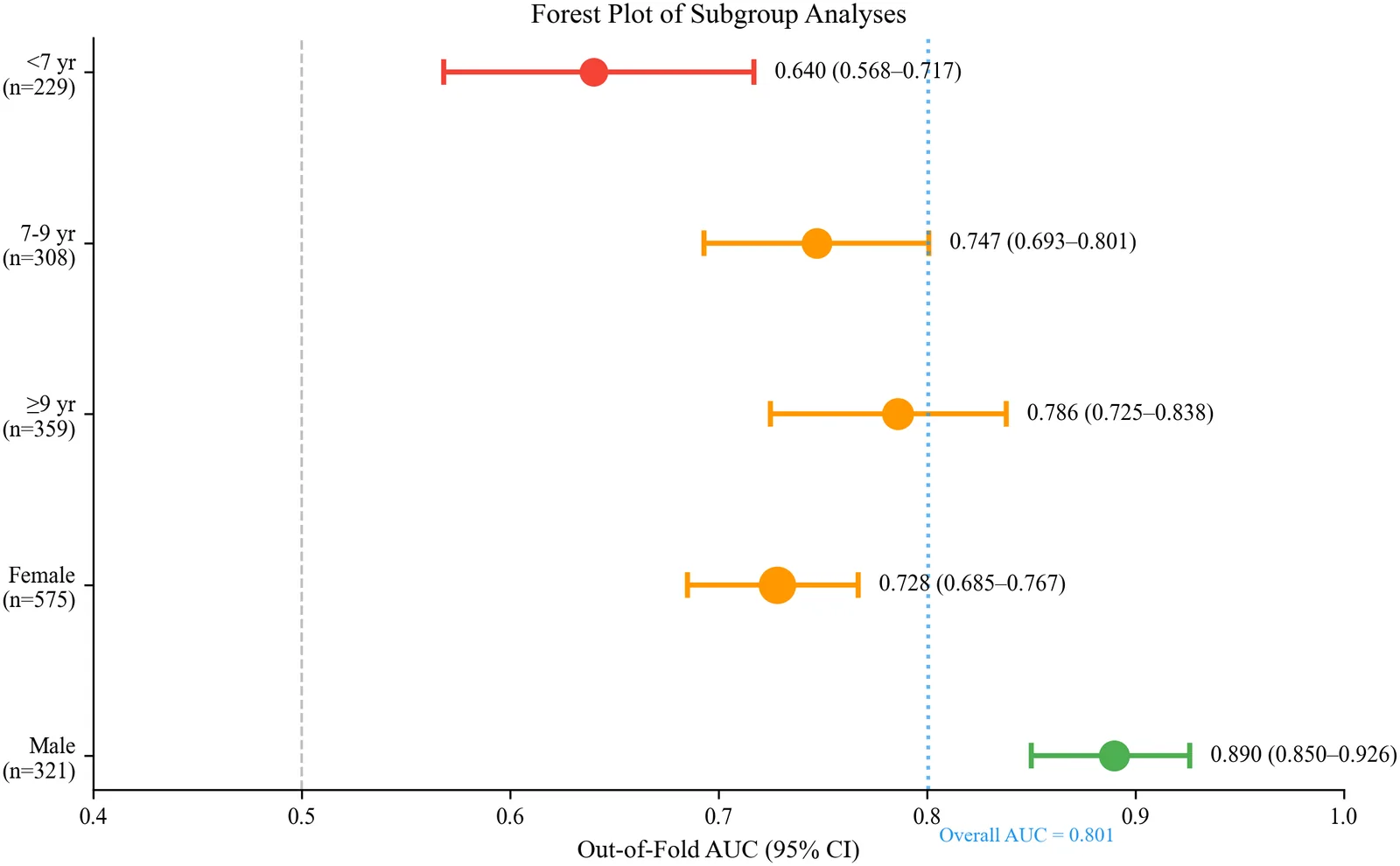
Subgroup analysis of out-of-fold AUC (95% CI) by age stratum and sex. Performance was highest in boys (0.890, 95% CI 0.850–0.926) and in children aged ≥9 years (0.786, 95% CI 0.725–0.838), and lowest in children aged <7 years (0.640, 95% CI 0.568–0.717). The 7–9 year group showed (0.747, 95% CI 0.693 -0.801) and girls (0.728, 95% CI 0.685 -0.767).

### 3.12 Sensitivity and stratified analyses

Propensity score matching on age and sex (1:1, caliper = 0.2 SD) yielded a matched cohort of 686 children in which the PV-only AUC was 0.610, confirming that the PV–HPGA association is attenuated but not eliminated after rigorous balancing on developmental stage. Simple categorical risk scores demonstrated clinically usable performance without any model fitting: a PV tertile score alone achieved an AUC of 0.693 (95% CI 0.659–0.727), an IGF-1 tertile score alone 0.710 (95% CI 0.676–0.743), and the two scores combined 0.748 (95% CI 0.716–0.779)—approaching the performance of the full multivariable model. In the diagnostically challenging subgroup with borderline IGF-1 values (124–245 ng/mL, the cohort IQR; n = 449), where the clinical model achieved an AUC of 0.682, adding PV raised the AUC to 0.688 (ΔAUC +0.006), twice the whole-cohort increment. Alternative outcome definitions yielded consistent conclusions (full sensitivity analyses in **Supplementary Tables S1**-**S3**), and the multi-algorithm comparison confirmed logistic regression’s adequacy.

### 3.13 Dual-granularity risk stratification and illustrative clinical application profiles

Following the dual-level classification strategy used in recent imaging-based prediction studies, our results support two complementary output granularities. First, a binary screen using the 203.9 mm³ Youden-optimal PV cutpoint: its classification matrix against the GnRH stimulation reference standard is shown in **Table 8**. Of 896 children, 599 (66.9%) were correctly classified; false negatives (n = 210) predominated over false positives (n = 87), an error profile consistent with a rule-out-oriented screening test in which minimizing missed activations is prioritized over avoiding false alarms. Second, a three-tier stratification anchored to the extreme PV deciles (≤115, 115–335, ≥335 mm³) brackets children into low (~29%), intermediate, and high (~92%) activation-risk categories. As in the three-tier model comparison framework (imaging-only vs clinical-only vs combined), the imaging-only PV test retained moderate standalone discrimination (AUC 0.730) whereas the combined model did not significantly outperform the clinical-only model (ΔAUC +0.003), directing clinical utility toward threshold-based stratification rather than incremental model discrimination. **Figure 7** presents three illustrative clinical application profiles constructed from cohort risk strata, mapping each PV tier to its observed activation prevalence and a concrete management suggestion; these profiles summarize cohort-level findings and are not individual patient data.

**Table 8:** Classification matrix of the 203.9 mm³ pituitary volume cutpoint against the GnRH stimulation reference standard (n = 896).

PV cutpoint result	HPGA+ (reference)	HPGA– (reference)	Total
PV ≥ 203.9 mm³ (test +)	343 (TP)	87 (FP)	430
PV < 203.9 mm³ (test −)	210 (FN)	256 (TN)	466
Total	553	343	896

Cell counts were derived from the reported sensitivity (62.0%), specificity (74.6%), and reference group sizes (553 activated, 343 non-activated); the positive and negative predictive values computed from the derived counts (79.8% and 54.9%) match the reported values. Overall accuracy 66.9% (599/896). False negatives (n = 210) predominate over false positives (n = 87), an error structure appropriate for a rule-out-oriented screening cutpoint. TP, true positive; FP, false positive; FN, false negative; TN, true negative.

**Figure 7:**
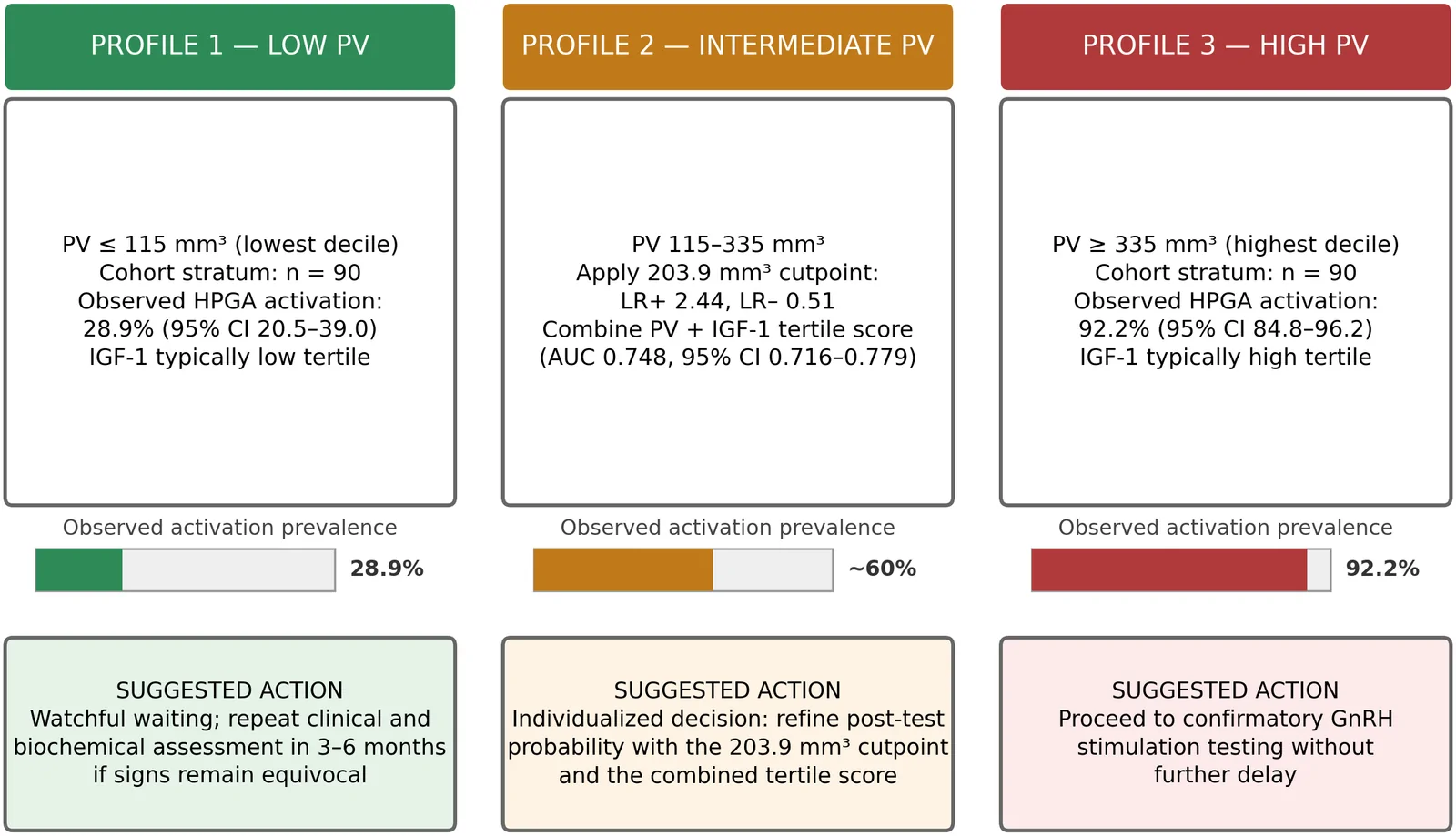
Illustrative clinical application profiles derived from cohort risk strata. Three representative profiles map pituitary volume (PV) tiers to observed HPGA activation prevalences and suggested management: low PV (≤115 mm³, lowest decile), intermediate PV (115–335 mm³, refined by the 203.9 mm³ cutpoint and the PV + IGF-1 tertile score), and high PV (≥335 mm³, highest decile). Profiles summarize cohort-level findings and are not individual patient data.

To facilitate clinical interpretation in centers where radiology reports provide pituitary height (PH) rather than volume, the PH values corresponding to each PV stratum were derived from the cohort (**Supplementary Table S6**; PV and PH were moderately correlated, Spearman ρ = 0.486): PV ≤115 mm³ corresponded to a median PH of 3.2 mm (IQR 2.8–3.7; activation 29.2%); PV 115–203.9 mm³ to PH 4.0 mm (IQR 3.5–4.5; activation 48.9%); PV 203.9–335 mm³ to PH 4.4 mm (IQR 3.9–5.2; activation 76.3%); and PV ≥335 mm³ to PH 5.0 mm (IQR 4.4–6.0; activation 92.3%). Thus, the low-, intermediate-, and high-risk PV tiers correspond approximately to PH <3.5 mm, 3.5–5.0 mm, and >5.0 mm, respectively. The PH-only extreme-decile gradient was attenuated relative to PV (42.4% activation at PH ≤3.0 mm vs 85.3% at PH ≥5.6 mm), consistent with the lower univariate discrimination of PH (AUC 0.658 vs 0.730 for PV); volumetric assessment is therefore preferred where feasible.

## 4 Discussion

### 4.1 Summary of key findings

In this large single-center cohort of 896 children undergoing both pituitary MRI and GnRH stimulation testing, we found that: (1) PV functions as a permissive morphological threshold of HPGA activation: only 28.9% (95% CI 20.5–39.0) of children in the lowest PV decile were activated versus 92.2% (95% CI 84.8–96.2) in the highest decile, with an OR of 4.38 (95% CI 2.60–7.36) between the model-predicted probabilities at the 90th and 10th percentiles (the OR per IQR, comparing the 75th vs 25th percentile, was 2.04, 95% CI 1.59–2.62); (2) PV is the second most important predictor in the model (Shapley +0.065 AUC, 22.2%; permutation importance second only to age) and carries unique, non-redundant information (LMG 20.4% of pseudo-R²; PV residual β = 0.295, 95% CI 0.119–0.472, P = 0.001); (3) PV provides actionable risk stratification, with each IQR increase (106 mm³) doubling activation odds (OR 2.04, 95% CI 1.59–2.62) and high-tertile membership conferring threefold adjusted odds (OR 3.11, 95% CI 2.02–4.78); (4) statistical mediation analysis generates the hypothesis that IGF-1 mediates approximately 37% of the PV–HPGA association; (5) the 95% CI of the incremental ΔAUC (+0.003; 95% CI −0.007 to +0.013) excludes a clinically meaningful improvement (≥0.05), and formal power analysis shows that approximately 25,360 children would be needed to confirm an effect of this size; and (6) the apparent univariate discrimination of PV (AUC 0.730) is largely explained by developmental confounding, with an age-sex-adjusted z-score AUC of 0.592.

### 4.2 PV as a strongly permissive morphological threshold

The most striking and clinically novel finding of this study is the threshold pattern at the extremes of the PV distribution. HPGA activation was rare when the gland was very small (28.9% in the lowest decile) and nearly universal when it was large (92.2% in the highest decile). This pattern is exactly what would be predicted if a minimum pituitary size—reflecting a critical mass of gonadotrophs capable of sustaining pulsatile LH output—were a strongly permissive morphological threshold for axis activation: without sufficient pituitary growth, HPGA activation is physiologically improbable, whereas a large gland permits but does not compel activation. The asymmetry of the distribution reinforces this interpretation: among non-activated children, 71.7% had PV below the median, indicating that a small gland effectively vetoes activation, whereas among activated children, 63.1% had PV above the median, indicating that a large gland permits but does not compel activation. This is the signature of a permissive, not strictly necessary, condition. Physiologically, the threshold concept is consistent with the classic morphometric literature: Elster et al. (1990) (7) and Kato et al. (2002) (8) showed that the pituitary enlarges physiologically during puberty as gonadotrophs hypertrophy under hypothalamic stimulation, so a gland that has not undergone this enlargement is anatomically unprepared to sustain axis activation. Clinically, this reframes PV from a ‘weak incremental predictor’ into a morphological checkpoint that contextualizes all downstream testing: a borderline IGF-1 value in a child with a very small pituitary carries a different meaning than the same value in a child with a large gland.

### 4.3 Relative importance and unique contribution of PV

Our three complementary decomposition frameworks directly address that: PV is the second most important predictor of HPGA activation, behind only age. Shapley decomposition attributed 22.2% of the model’s total AUC gain to PV—more than IGF-1 (21.4%), the most widely used biochemical surrogate. Permutation importance confirmed that PV (0.058 ± 0.007) and IGF-1 (0.060 ± 0.006) contribute statistically equivalent discrimination, both far below age but well above IGFBP-3 and sex. LMG partitioning attributed 20.4% of the model pseudo-R² to PV, exceeding IGF-1 (3.7%), IGFBP-3 (8.9%), and sex (4.5%) individually. Most importantly, the PV residual—the component of PV that cannot be predicted from age, sex, IGF-1, or IGFBP-3—remained significantly associated with HPGA activation (β = 0.295, SE 0.090, 95% CI 0.119–0.472, P = 0.001) and retained an AUC of 0.547 (95% CI 0.509–0.586) on its own. PV therefore contributes genuinely unique anatomical information that is not redundant with the biochemical and developmental variables already in the model. This uniqueness, combined with the threshold pattern, distinguishes PV from a purely collinear surrogate.

### 4.4 Mediation pathway: hypothesis-generating evidence

The statistical mediation analysis is consistent with a biologically plausible pathway in which pituitary enlargement reflects increased somatotroph and gonadotroph activity, raising systemic IGF-1, which in turn associates with HPGA activation. IGF-1 is known to rise during puberty and to stimulate gonadotropin secretion (17, 28–30), and the pituitary gland is both a source and a target of IGF-1 signaling. That 37.4% of the PV effect is statistically mediated through IGF-1 while the majority (approximately 62%) remains direct is mechanistically interesting: it suggests that pituitary size indexes gonadotroph capacity largely independently of the somatotroph–IGF-1 axis, consistent with the morphological-threshold interpretation. We emphasize, however, that Baron-and-Kenny-style mediation with a logistic outcome is subject to non-collapsibility (hence our dual reporting of 37.4% and 35.1%), relies on the strong assumption of no unmeasured exposure–mediator, exposure–outcome, or mediator–outcome confounding, and cannot establish temporal ordering in cross-sectional data (20–22). The finding should therefore be read as hypothesis-generating: it nominates IGF-1 as a candidate mechanistic link for prospective, longitudinal confirmation rather than confirming a biologically validated pathway.

### 4.5 Clinical translation: from incremental value to clinical decision support

A central, honest finding of this study is that PV adds little discriminative information to a well-specified clinical model (ΔAUC +0.003). Age, basal gonadotropins, and IGF-1 already capture most of the discriminative information that correlates with PV. Within PV tertiles, the continuous measurement adds nothing (ΔAUC ≤ 0 in all three strata). However, the power analysis fundamentally reframes this finding: with N = 896, the study had only ~5% power to detect a ΔAUC of 0.003. Approximately 25,360 children would be needed for 80% power—a sample size that exceeds most published pediatric endocrine imaging studies. The appropriate interpretation is therefore not that PV definitively lacks incremental value, but rather that its incremental value—which is likely small—could not be statistically confirmed given the available sample. This distinction between ‘absence of evidence’ and ‘evidence of absence’ is critical and has been underappreciated in the imaging biomarker literature. The DCA results reinforce this interpretation: the direction of ΔNB was positive at the clinically most relevant thresholds (0.30–0.40 and 0.60), though all confidence intervals included zero. Reassuringly, the incremental signal was larger exactly where diagnostic uncertainty is greatest: ΔAUC +0.027 in the 7–9 year group and +0.006 in borderline IGF-1 cases, both exceeding the whole-cohort estimate.

To translate these findings into actionable guidance, we outline three concrete clinical scenarios anchored to exact PV thresholds.

Scenario 1 (low PV): a child with PV ≤ 115 mm³ (lowest decile) has an observed activation prevalence of only 28.9% (95% CI 20.5–39.0); if the IGF-1 level is borderline and pubertal signs are equivocal, watchful waiting with repeat clinical and biochemical assessment in 3–6 months is a reasonable alternative to immediate GnRH stimulation testing.

Scenario 2 (high PV): a child with PV ≥ 335 mm³ (highest decile) has an activation prevalence of 92.2% (95% CI 84.8–96.2); with concordant clinical features, proceeding directly to confirmatory stimulation testing avoids diagnostic delay.

Scenario 3 (intermediate PV with borderline biochemistry): for the substantial fraction of children whose values fall between these extremes—particularly those aged 7–9 years or with borderline IGF-1 (124–245 ng/mL)—the 203.9 mm³ cutpoint provides a practical anchor: a PV above this value carries an LR+ of 2.44, meaningfully raising the post-test probability of activation, whereas a PV below it carries an LR− of 0.51 (DOR 4.78; NNS 2.73). The combined PV-plus-IGF-1 tertile score (AUC 0.748, 95% CI 0.716–0.779) can be computed at the bedside without any model fitting.

Integration into existing workflows is straightforward, and is strictly contingent on guideline-based MRI indications: current guidelines recommend cranial MRI for all boys with CPP, all girls younger than 6 years with CPP, and girls aged 6 years or older with rapidly progressive sexual development or neurological abnormalities, to exclude intracranial lesions; pituitary imaging is not mandatory for the diagnosis of precocious puberty per se, and MRI should not be ordered solely to obtain PV. Where pituitary MRI has already been performed for these indications, the ellipsoid measurement adds less than two minutes to the radiology read and can be embedded as a structured-report field at negligible incremental cost. From a cost-effectiveness perspective, the intervention is dominant where MRI is already indicated: even a modest reduction in unnecessary GnRH stimulation tests would offset the trivial incremental measurement cost many times over. Conversely, we emphasize that MRI should not be ordered solely for the purpose of obtaining PV measurements. These thresholds are derived from a single-center cohort and necessitate external validation before widespread adoption. Furthermore, PV must always be interpreted in conjunction with clinical examination and biochemical testing, rather than as a substitute.

### 4.6 Strengths and limitations

Strengths of this study include the large sample size (N = 896), blinded and reproducible volumetry with high inter-reader reliability, rigorous internal validation with repeated cross-validation and OOF estimates, the three-layer statistical framework (relevance → uniqueness → utility) that provides a logical, clinically accessible analytic structure, prespecified incremental-value testing with the DeLong method (18), three convergent relative-importance frameworks (Shapley (24), permutation, LMG (25)) triangulating PV’s contribution, decision curve analysis with bootstrap confidence intervals as recommended by Vickers and Elkin (26, 27), transparent dual reporting of mediation proportions, and—critically—a formal power analysis that distinguishes absence of evidence from evidence of absence. The multi-algorithm comparison and propensity score-matched sensitivity analysis further support the robustness of the primary findings. The explicit extreme-value analysis at the distributional extremes, the residual-based isolation of PV’s unique information, and the simple tertile risk scores provide a nuanced understanding of where and how PV contributes diagnostic information.

Several limitations merit emphasis. First and most important, this was a retrospective, single-center study: the cohort was assembled from children referred to a tertiary center who underwent both pituitary MRI and GnRH stimulation testing, and our performance estimates derive from internal cross-validation rather than external validation. The absence of external, prospective, multi-center validation is a major limitation, and the reported AUCs are likely optimistic relative to performance in independent cohorts. Second, the design invites selection and spectrum bias: children referred for pituitary MRI may differ systematically from the broader population of children with early pubertal signs—for example, toward more severe, atypical, or diagnostically ambiguous presentations—so the observed PV distribution and activation prevalences may not transport to primary-care or community settings; verification bias is also possible because only children who proceeded to stimulation testing were included. Third, generalizability is limited by imaging and assay heterogeneity: Absolute volumes may differ across scanner vendors, field strengths, sequences, and measurement conventions; normative PV also varies with ethnicity and nutritional status, and our HPGA definition reflects one specific LH assay and threshold. The decile and cutpoint values we report (115, 203.9, 335 mm³) should therefore be recalibrated locally before clinical use. Fourth, the cross-sectional design precludes causal inference regarding the PV–HPGA pathway; the mediation analysis is associational and hypothesis-generating only, as it cannot rule out unmeasured confounding of the exposure–mediator, mediator–outcome, or exposure–outcome relationships, and the PV→IGF-1→HPGA pathway remains a hypothesis pending longitudinal and interventional evidence. Fifth, MRI availability and cost limit applicability to settings where pituitary imaging is routine. Sixth, performance was poor in children under 7 years, restricting applicability in that age group. Seventh, elevated VIF values for some predictors (e.g., height, BMI, age) reflect the expected collinearity among developmental variables; while the logistic regression coefficient estimates remain unbiased, their standard errors may be inflated. Eighth, the Hosmer-Lemeshow test indicated modest miscalibration (χ² = 21.48, P = 0.006), predominantly in the intermediate predicted-probability range (0.40–0.70), likely reflecting unmodeled non-linear relationships between PV and HPGA activation; restricted cubic splines or generalized additive models may better capture these non-linearities but were not pursued because the primary aim was to estimate incremental value within a simple, clinically transparent logistic framework. The Brier score of 0.178 nonetheless indicates acceptable overall calibration, and because calibration-in-the-large does not affect rank-based discrimination, this miscalibration does not alter any AUC- or ΔAUC-based conclusion. Ninth, residual confounding by unmeasured developmental factors (e.g., Tanner stage granularity, bone age) cannot be excluded, and the extreme-value analysis, while striking, is descriptive at the extremes where subsample sizes were modest (n = 90 per decile). Tenth, the morphological-threshold concept itself has limitations: PV is not a strictly necessary condition in the logical sense—28.9% of children with very small glands still showed activation—and it should be interpreted as a strongly permissive morphological threshold in practice rather than an absolute gate. Clinicians should therefore be aware that a very small PV does not completely rule out HPGA activation, although it substantially reduces the pre-test probability; conversely, a large PV permits but does not prove activation. Finally, all findings are based on traditional regression-based statistics; although we compared against random forest, gradient boosting, SVM, and KNN and found no material improvement, more sophisticated deep-learning approaches (e.g., 3D convolutional neural networks applied directly to pituitary MRI) have not yet been evaluated and could yield additional predictive value in future work. Accordingly, external validation in independent, non-tertiary cohorts is required before the model can be adopted in community or primary-care settings; additionally, the age- and sex-adjusted PV z-score was standardized against internal cohort norms; its modest discrimination (AUC 0.592) may partly reflect this in-sample standardization, and z-scores referenced to external normative imaging datasets could perform differently.

Eleventh, because boys with organic central nervous system lesions were excluded, the PV threshold derived in this study is applicable to boys with early pubertal manifestations in whom the gonadal axis is either activated or not yet activated, and should not be used for screening for pathological CPP in boys; any boy diagnosed with CPP requires cranial MRI to rule out intracranial lesions regardless of PV. Twelfth, 60 children aged ≥12 years (7 girls and 53 boys) fell outside the strict Chinese definition of precocious puberty; they were retained because they represent the full spectrum of referrals for HPGA axis evaluation, and sensitivity analyses after their exclusion confirmed the robustness of the main findings.

### 4.7 Clinical significance and future research directions

The present findings reposition pituitary volume from an underutilized morphometric observation into a clinically actionable morphological threshold that can be evaluated at minimal cost using MRI scans already acquired in many diagnostic workflows. Three implications merit emphasis. First, in diagnostically ambiguous clinical scenarios—children with equivocal pubertal signs, borderline IGF-1 values, or intermediate clinical risk scores—PV provides a non-invasive, radiation-free, and already-available data point that can either raise or lower the post-test probability of HPGA activation in a clinically meaningful way (LR + 2.44, LR− 0.51). Second, by reframing PV as a morphological threshold rather than a continuous diagnostic test, we offer pediatric endocrinologists a more clinically intuitive mental model: very small glands make activation unlikely and argue for watchful waiting; very large glands make activation highly likely and argue for prompt confirmatory testing; intermediate glands should be interpreted alongside clinical and biochemical data. Third, the explicit demonstration that incremental ΔAUC of +0.003 could not be statistically confirmed in a cohort of 896 children (with ~5% power) should recalibrate the expectations of clinicians and researchers: the standard threshold of P < 0.05 is uninformative in this context, and substantially larger samples or alternative evaluation frameworks (e.g., decision-curve net benefit, clinical utility at prespecified thresholds) are needed to assess the true incremental value of imaging biomarkers in endocrinology.

Several research directions emerge. External, prospective, multi-center validation of the proposed PV thresholds (115, 203.9, 335 mm³) and the combined PV-plus-IGF-1 tertile risk score is the most urgent priority. Longitudinal studies that follow children with very small or very large PV over time would clarify whether PV trajectories, rather than single-time-point measurements, carry additional prognostic information. Finally, integration of pituitary MRI with radiomics and deep-learning approaches—building on our team’s earlier radiomics work (12)—may further improve the diagnostic and prognostic utility of pituitary imaging in this population, particularly in the diagnostically challenging 7–9 year age group where the largest incremental signal was observed.

## 5 Conclusions

Pituitary volume is a permissive morphological threshold of HPGA activation in children: although not a strictly necessary condition, below a critical gland size activation is physiologically improbable enough to be clinically informative (28.9% vs 92.2% activation at the extreme deciles; OR 4.38, 95% CI 2.60–7.36; OR per IQR 2.04). PV is the second most important predictor (Shapley 22.2%) and carries unique, non-redundant information (LMG 20.4% of pseudo-R²; PV residual β = 0.295, P = 0.001), and mediation analysis generates the testable hypothesis that approximately 37% of the association is mediated by IGF-1. The apparent standalone discrimination of PV largely reflects developmental confounding (age-sex-adjusted AUC 0.592), and its incremental value over clinical parameters is small (ΔAUC +0.003, 95% CI −0.007 to +0.013). Accordingly, PV must not be used as an independent test to confirm or exclude HPGA activation: the GnRH stimulation test remains the reference standard, and pituitary MRI should be performed according to current guidelines for the exclusion of intracranial lesions—not solely to obtain PV. When such MRI has already been acquired, PV serves as a complementary morphological checkpoint that contextualizes downstream hormone interpretation, most valuable in the 7–9 year age group and in borderline IGF-1 cases. Prospective external validation and adequately powered multi-center studies are warranted.

## Data Availability

The data analyzed in this study is subject to the following licenses/restrictions: The de-identified datasets generated and analyzed during the current study, are available from the corresponding authors on reasonable request, subject to institutional data sharing policies. Requests to access these datasets should be directed to Dong Liu, liudongshole@163.com.
