# Supplementary material for: Pituitary volume as a permissive morphological threshold for hypothalamic-pituitary-gonadal axis activation in children: a comprehensive diagnostic prediction study with relative importance decomposition
Source: Front Endocrinol (Lausanne). 2026 Oct 22;17:1981603. doi: 10.3389/fendo.2026.1981603 (PMC13735225; doi:10.3389/fendo.2026.1981603)
Supplement: Supplementary file 1 [file SupplementaryFile1.docx]

**Supplementary Material**

Article ID: 1981603
Title: Pituitary volume as a permissive morphological threshold for HPGA activation in children
Corresponding Author: Jun Lu ([lujun@tjh.tjmu.edu.cn](mailto:lujun@tjh.tjmu.edu.cn)) Dong Liu(liudongshole@163.com)
Date: 2026-10-16

*This supplementary material contains S1 Methods and S1–S6 Tables. All files are provided as a single document without line numbers, in accordance with the journal's submission requirements.*

================================================================================

**S1 Methods**

Detailed methodological descriptions, including the power-analysis formulas, bootstrap procedures, and Shapley/LMG decomposition algorithms, are provided in this section.

**Power Analysis Formulas:**

The power calculation for the incremental AUC was performed using the formula for comparing two correlated ROC curves, accounting for the correlation between predictions from the full and clinical-only models.

**Bootstrap Procedures:**

Bootstrap confidence intervals (95% CI) were computed using 1000 resamples with replacement. The percentile method was used to derive confidence limits.

**Shapley/LMG Decomposition Algorithms:**

Shapley value decomposition was implemented using the 'iml' package in R, which computes the average marginal contribution of each predictor across all possible orderings. LMG (Lindeman-Merenda-Gold) variance partitioning was performed using the 'relaimpo' package to decompose the model R² into non-negative per-predictor contributions.

================================================================================

**S1 Table. Propensity Score Matching Results**

Matching performed on age and sex (1:1, caliper = 0.2 SD). Matched cohort: n = 686 (343 activated, 343 non-activated). PV-only AUC in matched cohort: 0.610 (95% CI 0.568–0.652). The PV–HPGA association is attenuated but not eliminated after rigorous balancing on developmental stage.

================================================================================

**S2 Table. Alternative Outcome Definitions**

Alternative outcome definitions tested:
1. LH peak ≥ 5.0 IU/L: AUC 0.730 (primary definition)
2. LH peak ≥ 4.0 IU/L: AUC 0.728
3. LH peak ≥ 6.0 IU/L: AUC 0.725
All definitions yielded consistent conclusions regarding the incremental value of PV.

================================================================================

**S3 Table. Sensitivity Analyses**

Sensitivity analyses performed:
1. Complete-case analysis (excluding missing data): AUC 0.731
2. Multiple imputation: AUC 0.729
3. Excluding outliers (PV > 3 SD from mean): AUC 0.728
All sensitivity analyses confirmed the robustness of the primary findings.

================================================================================

**S4 Table. Clinical characteristics of boys (n = 321) stratified by HPGA activation status**

| Characteristic | HPGA+ (n = 183) | HPGA− (n = 138) |
| --- | --- | --- |
| Age (years), median (IQR) | 11.16 (10.20–12.11) | 6.70 (5.12–8.40) |
| Pituitary volume (mm³), median (IQR) | 230.6 (182.0–296.7) | 151.8 (115.5–187.7) |
| Pituitary height (mm), median (IQR) | 4.2 (3.8–5.0) | 3.7 (3.0–4.2) |
| IGF-1 (ng/mL), median (IQR) | 185 (148–252) | 103 (74–139) |

**HPGA activation prevalence by age:**

| Age Group | Prevalence |
| --- | --- |
| 3–<6 y | 12/65 (18.5%) |
| 6–<7 y | 4/30 (13.3%) |
| 7–<9 y | 10/41 (24.4%) |
| 9–<12 y | 106/132 (80.3%) |
| ≥12 y | 51/53 (96.2%) |

================================================================================

**S5 Table. Sex-specific age-stratified analysis (age strata aligned with Chinese diagnostic criteria)**

| Stratum | n | HPGA+, n (%) | PV median (IQR), mm³ | PV HPGA+ median |
| --- | --- | --- | --- | --- |
| Girls 3–7.5 y | 188 | 91 (48.4%) | 170.4 (124.7–215.1) | 180.5 |
| Girls 7.5–9 y | 213 | 137 (64.3%) | 211.6 (168.3–266.4) | 226.8 |
| Girls 9–10 y | 109 | 89 (81.7%) | 243.3 (190.3–332.9) | 250.5 |
| Girls ≥10 y | 63 | 51 (81.0%) | 240.4 (203.9–292.7) | 247.9 |
| Boys 3–9 y | 136 | 26 (19.1%) | 141.4 (111.7–170.6) | 159.8 |
| Boys 9–11 y | 78 | 54 (69.2%) | 206.9 (171.6–263.0) | 230.5 |
| Boys 11–13 y | 88 | 85 (96.6%) | 243.1 (206.6–315.1) | 241.6 |
| Boys ≥13 y | 19 | 18 (94.7%) | 278.0 (253.2–365.9) | 297.9 |

================================================================================

**S6 Table. Corresponding pituitary height for each pituitary volume (PV) stratum**

| PV stratum (mm³) | n | Corresponding pituitary height, median (IQR), mm | HPGA activation prevalence | Clinical risk tier |
| --- | --- | --- | --- | --- |
| PV ≤115 (lowest decile) | 89 | 3.2 (2.8–3.7) | 29.2% | Low risk (PH <3.5 mm) |
| PV 115–203.9 | 378 | 4.0 (3.5–4.5) | 48.9% | Intermediate risk (PH 3.5–5.0 mm) |
| PV 203.9–335 | 338 | 4.4 (3.9–5.2) | 76.3% | Intermediate risk (PH 3.5–5.0 mm) |
| PV ≥335 (highest decile) | 91 | 5.0 (4.4–6.0) | 92.3% | High risk (PH >5.0 mm) |

*Note: PV and pituitary height (PH) were moderately correlated (Spearman ρ = 0.486). The PH-only extreme-decile gradient was attenuated relative to PV (42.4% activation at PH ≤3.0 mm vs 85.3% at PH ≥5.6 mm), consistent with the lower univariate discrimination of PH (AUC 0.658 vs 0.730 for PV); volumetric assessment is preferred where feasible.*
